# The True Cost of Greenhouse Gas Emissions: Analysis of 1,000 Global Companies

**DOI:** 10.1371/journal.pone.0078703

**Published:** 2013-11-12

**Authors:** Nagisa Ishinabe, Hidemichi Fujii, Shunsuke Managi

**Affiliations:** 1 Department of Agricultural Economics, Purdue University, West Lafayette, Indiana, United States of America; 2 Graduate School of Environmental Studies, Tohoku University, Sendai, Japan; 3 Institute for Global Environmental Strategies, Hayama, Kanagawa, Japan; Universidad Veracruzana, Mexico

## Abstract

This study elucidated the shadow price of greenhouse gas (GHG) emissions for 1,024 international companies worldwide that were surveyed from 15 industries in 37 major countries. Our results indicate that the shadow price of GHG at the firm level is much higher than indicated in previous studies. The higher shadow price was found in this study as a result of the use of Scope 3 GHG emissions data. The results of this research indicate that a firm would carry a high cost of GHG emissions if Scope 3 GHG emissions were the focus of the discussion of corporate social responsibility. In addition, such shadow prices were determined to differ substantially among countries, among sectors, and within sectors. Although a number of studies have calculated the shadow price of GHG emissions, these studies have employed country-level or industry-level data or a small sample of firm-level data in one country. This new data from a worldwide firm analysis of the shadow price of GHG emissions can play an important role in developing climate policy and promoting sustainable development.

## Introduction

Our study focuses on the true cost of emissions reduction at the firm level by computing the evidence-based shadow price of greenhouse gas (GHG) emissions by company, which is estimated by a production function approach using GHG data. The study covers 1,024 major companies of 17 industries in 37 countries over the period from 2002 to 2009. This survey allows us to observe the patterns of the shadow price of GHG emissions among firms and sectors over many years and to examine efficient and effective pathways to transform our socio-economic systems into sustainable systems.

To ameliorate the effects of climate change, each country in the world is currently advancing research on GHG reduction methods and the costs of such reductions [1]–[2]. Although many previous studies have focused on the shadow price of GHG, most of these studies are focused on future shadow prices at the national and global levels based on projected scenarios and do not actually calculate the current shadow price or calculate the shadow price at the company level^3^. Therefore, when regulations pertaining to the total quantity of GHG emissions are introduced, the degree of impact on each company in each country is uncertain. Consequently, it is difficult to allay the concerns of companies in any country as to whether they will suffer an adverse effect on their international competitiveness when carbon constraints are imposed. As a result, companies and countries are currently not assuming obligations to reduce the total quantity of GHG emissions. Therefore, this study determines the present shadow price and the effect of market competitiveness in terms of GHG emissions at the company level as well as the degree of impact on each company under carbon constraints and on its international competitiveness when reductions in the total quantity of GHG emissions are introduced.

## Methods

The shadow price that is calculated by a production function is equivalent to the revenue to be sacrificed when a company is forced to reduce one ton of GHG emissions. A lower shadow price signals that it is relatively less expensive to reduce GHGs. The shadow price captures the holistic price of GHG emissions for a company by considering both technological advancements and operational efficiencies (e.g., switching off lights and ensuring the optimal use of materials) by employing all firm-level data. As a result, the shadow price is distinct from technology-based abatement costs or GHG intensity, which captures the cost of only a single technology or the ratio of GHG emissions and sales.

The economic valuation method for handling environmentally undesirable outputs using the directional distance function (DDF) as a nonparametric approach was developed by [3]–[4]. Following [3], we can estimate q, the economic value of an environmentally undesirable output (shadow price), using the measure below.

We denote 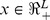, 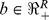 and 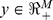 as the vectors of inputs, environmental output (or undesirable output), and market outputs (or desirable output), respectively, and we then define the production technology as follows:
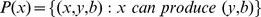
(1)


The inefficiency D(*x*, *y*, *b*| *g_x_*, *g_y_*, *g_b_*) of the production units in P(*x*) for each of the firms in this study is defined with the distance from the production frontier consisting of the efficient production units as follows:
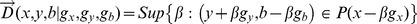
(2)where g_x_, g_y_, and g_b_ denote the non-negative directional vectors of the input, the desirable output, and the undesirable output, respectively. From the above definition, equation (3) is determined to be valid.



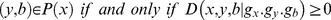
(3)Under a perfect competitive market, the prices of market goods, *p*, and the prices of undesirable goods, *q*, are assumed to be *p*>0 and *q* <0, respectively. If the aggregate economic value of the output for each production unit is given by R(*x*, *p*, *q*), then the specific combination of *y* and *b* to maximize *py+qb,* (y*,b*)εP(*x*) for given prices of *p* and *q* exists in the production possibility set. Therefore, R(*x*, *p*, *q*) can be expressed as follows:
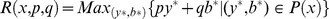
(4)Here, with equation (3), *R*(*x*, *p*, *q*) becomes the following:
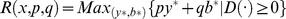
(5)where D(*x, y, b*| *g_x_, g_y_, g_b_*) is expressed as D(⋅). Given that equation (5) is formed for any pairs (*y, b*) P(x), the relation (y*,b*) = (*y*+D(⋅)×*g_y_*, *b*+D(⋅)×*g_b_*) can replace equation (5). Thus, 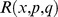 is obtained as follows:



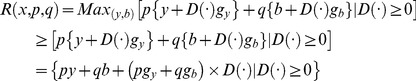
(6)Furthermore,
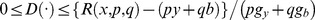
(7)


Simultaneously, because *R*(*x*, *p*, *q*) is function of *p* and *q* (hence, for any pairs (*y*,*b*) ε *P*(*x*)), a certain *p* and *q* exists to satisfy the following relationship:
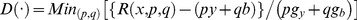
(8)


Executing the partial differentiation for both sides of equation (8) with respect to *b* and *y*, equations (9) and (10), respectively, can be derived:
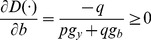
(9)

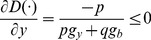
(10)


Equation (9) describes the extent of the increase in inefficiency *D*(⋅) while emitting an additional environmental undesirable output by one unit marginally. Similarly, equation (10) describes the extent of the decrease in inefficiency *D*(⋅), while increasing the additional market output by one unit marginally. Combining equations (9) and (10) then results in equation (11):
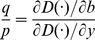
(11)


Therefore, by simply solving equation (11) with *q*, the economic value of the environmentally undesirable output is defined as follows:
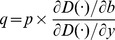
(12)


Here, the economic value of the market output can be normalized as *p  = 1* if the market output variable consists of monetary data; thus, the economic value *q* is regarded as the value of the environmentally undesirable good relative to the value of the market goods.

We can estimate the adjusted shadow price *q* ^adj^, the economic value of an environmentally undesirable output considering an inefficiency score, using the following measure:
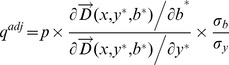
(13)where (y*, b*) is the intersecting point on the frontier curve with the directional vector of an inefficient province. The inefficiency factors 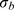 and 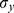 are defined as follows:



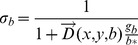
(14)

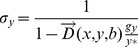
(15)


Here, we set the production function using input *x*, undesirable output *b*, and desirable output *y*. We assume desirable and undesirable outputs under a null-joint hypothesis; a company cannot produce a desirable output without producing undesirable outputs.
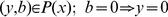
(16)


We also assume weak disposability. Weak disposability implies that the pollutant should not be considered freely disposable.
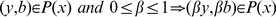
(17)


Under the null-joint hypothesis and weak disposability, this directional distance function can be computed for firm k by solving the following optimization problem:
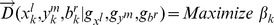
(18)

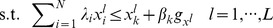
(19)

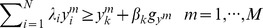
(20)

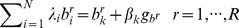
(21)

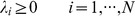
(22)where l, m, and r are the input, the desirable output, and the undesirable output, respectively; x is the input factor in the L × N input factor matrix; y is the desirable output in the M × N desirable output factor matrix; and b is the undesirable output factor in the R × N undesirable output matrix. In addition, g_x_ is the directional vector of the input factor, g_y_ is the directional vector of the desirable output factors, and g_b_ is the directional vector of the undesirable output factors. is the inefficiency score of the kth firm, and is the weight variable. To estimate the inefficiency score of all firms, the model must be independently applied N times for each firm. One objective of this study is to clarify the extent to which firms have improved their levels of productivity with respect to the CO_2_ emissions under consideration. We set the directional vector as (g_x_, g_y_, g_b_) = (0, y^m^, b^r^) to estimate the productivity change by applying the Luenberger productivity indicator. Under this directional vector setting, we obtain the following equation.

Objective function
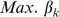
(23)


Restriction
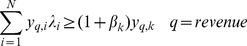
(24)

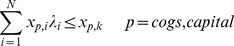
(25)

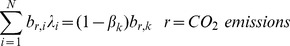
(26)

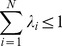
(27)

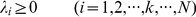
(28)


The model above estimates the inefficiency score by considering the extent to which a company can reduce its CO_2_ emissions and increase its revenue without increasing the cogs and capital. β represents the inefficiency score, and β >0 indicates that firm k inefficiently discharges CO_2_ emissions and loses revenue compared with the efficient firms that form the production frontier line curve.

Equation (27) representing the DDF model has applied the decreasing return-to-scale (DRS) assumption. The DDF model commonly requires the return-to-scale assumption. In this study, we apply the DRS assumption to avoid an infeasible calculation in the time series analysis. Note that assuming DRS does not eliminate the possibility of infeasible linear programming (LP) problems when weak disposability is imposed on bad outputs. When we model bad outputs in LP, the potential for infeasible LP problems results from imposing weak disposability on the undesirable outputs and from specifying an LP problem that uses period t reference technology with observations from period t+1. The variable return-to-scale (VRS) model tends to yield infeasible results more often than the DRS model when computing the productivity change because the VRS has stronger restrictions for solving a linear program. We confirmed with our models that the calculation of productivity changes under the VRS is infeasible. Therefore, we applied the DRS model in this study.

This study used the GHG emissions data and sales revenues by industry provided by the Trucost, and financial data provided by the Factset, respectively. Therefore, this study used data from these two companies, which is considered to ensure the highest level of quality of the data available at present.

The Trucost’s GHG emissions data is created by taking the quantity of GHG emissions disclosed by the businesses through environmental and financial reports supplemented by Trucost’s own processes of verification and modification. When verifying the data, Trucost confirms whether each company reports irregular figures by comparing the reported data with calculations of the plausible quantities of GHG emissions based on the quantity of energy used by the business in question, as well as by comparison with the GHG emissions of other companies in the same industry. Furthermore, if in this process a figure that could be considered irregular is discovered, interviews are held with representatives from the company concerned and corrections are made as required for the maintenance of the database. Since the data set is not a collection of unverified data released voluntarily by individual businesses, their data quality is better than data sets of Carbon Disclosure Project and similar sources, which is provided directly by the firms. At present, this is the most reliable database.

Regarding financial data, because this is reported by each company pursuant to the accounting standards of each country, and because each type of figure has undergone corporate auditing, reliable data that enables comparisons between companies can be obtained relatively easily. This study used the figures of the Factset, which is one of the largest business financial data firms in the world. Note that since accounting standards vary depending on the country, figures were used that were recompiled according to the Factset standard (a global integrated standard created by Factset). US dollars were adopted as the currency, and, after consolidating the exchange rates as of the end of the accounting year for each year, the figures were deflated to the year-2000 prices.

The research period covered in this study is in principle the eight-year period from 2002. The 17 industry sectors subject to analysis in this study are Automobiles & Parts, Basic Resources (including steel and paper industries), Chemicals, Oil & Gas, Utilities, Industrial Goods & Services, Construction & Materials, Personal & Household Goods, Telecommunications, Technology, Healthcare, Travel & Leisure, Retail, Food & Beverage, Financial, Real Estate, and Media services. For the classification of businesses into industry sectors, the ICB-Super Sector classification of the Financial Times of the UK and the London Stock Exchange which creates finance-related indexes, was referenced. In addition, retail, financial, and real estate services were integrated to ensure a sufficient quantity of data in each industry when running the model.

The data used comes under sales revenues, cost of goods sold (COGS), total assets, current assets, and GHG emissions (for the six gases listed for reduction in the Kyoto Protocol). For a figure for capital, fixed assets (total assets minus current assets) was chosen, and to express the (total) amount of labor and materials used, cost of goods sold was chosen. Cost of goods sold is the costs required to provide goods and services. For GHG emissions it was decided that all three scope categories would be included: Scope 1 (emissions in manufacturing processes), Scope 2 (emissions due to power usage among others), and Scope 3 (other emissions from commuting, business trips, and the supply chain).

This study applies the emission data for all the categories of Scope 1, 2, and 3 because of the following reasons; (1) using all three enables a more thorough assessment of the overall picture of each company’s GHG emissions and (2) since this study covers a broad range of industries, including IT, Media, Healthcare, and financial institutions, rather than specifically the manufacturing industry, limiting the scope would increase the number of cases that do not reflect the actual state of the company. However, standards for the supply chain data in Scope 3 are currently being debated in each industry, so the figures were deemed unworkable for comparisons between companies at present and it was decided to use only those for GHG emitted during the use of airplanes, railroads among others.

## Results

The average shadow price of GHG emissions for the observed sample of 1,024 companies in 37 countries worldwide is $10,414, and the median price is $4,189; these values are much higher than the corresponding values in any of the previous studies (Table 1). The reason for the higher shadow price is the use of the Scope 3 GHG emissions data in this study. This research implies that a firm would carry a high cost of GHG emissions if Scope 3 GHG emissions were the focus of discussions regarding corporate social responsibility. In fact, 10% of the observed companies may reduce emissions at a cost of $100 or less, approximately 30% at $1,000 or less, and 70% at $10,000 or less. Germany’s median shadow price is the highest at a price of $9,423 among the six countries with more than 200 observations: the United States, the United Kingdom, Japan, France, and China (including Hong Kong and Taiwan). France follows Germany at a price of $8,697, with the United Kingdom at $7,335, the United States at $3,340, China at $4,316, and Japan at $2,332 (see Figure S1 and Figure S2).

**Table 1:** Shadow price by country.

	Country	Average Shadow Price (U.S. $)	Median Shadow Price	Shadow Price (<100$)	Shadow Price (100 $– 1,000$)	Shadow Price (1,001$ – 10,000$)	# obs.
1	UNITED STATES	9,809	3,340	10%	19%	46%	2,321
2	UNITED KINGDOM	13,544	7,335	6%	13%	41%	1,119
3	JAPAN	6,626	2,332	8%	27%	47%	689
4	FRANCE	16,686	8,697	7%	8%	37%	319
5	CHINA+HK+TAIWAN	8,453	4,316	14%	22%	38%	277
6	GERMANY	15,433	9,423	6%	11%	36%	223
7	SWITZERLAND	11,003	5,579	10%	8%	47%	155
8	SWEDEN	14,074	7,086	8%	14%	34%	152
9	ITALY	11,406	5,780	15%	7%	43%	137
10	SPAIN	12,482	3,146	22%	18%	30%	133
11	CANADA	2,120	469	11%	59%	24%	118
12	NETHERLANDS	9,917	3,536	3%	11%	50%	109
13	FINLAND	13,088	1,986	18%	18%	30%	98
14	NORWAY	2,955	612	12%	45%	33%	67
15	SOUTH KOREA	5,739	3,216	3%	36%	43%	67
16	DENMARK	7,093	5,640	12%	17%	41%	58
17	INDIA	19,279	18,259	13%	16%	16%	45
18	BRAZIL	13,707	10,107	5%	15%	29%	41
19	MALAYSIA	4,253	582	38%	18%	25%	40
20	IRELAND	11,596	8,704	16%	8%	29%	38
21	AUSTRALIA	2,080	878	30%	24%	41%	37
22	BELGIUM	6,904	2,817	5%	22%	46%	37
23	PORTUGAL	9,417	7,830	29%	6%	26%	35
24	GREECE	5,743	5,103	11%	4%	70%	27
25	SINGAPORE	6,610	4,115	0%	13%	67%	24
26	AUSTRIA	2,815	685	41%	9%	45%	22
27	THAILAND	69	18	86%	14%	0%	21
28	MEXICO	5,830	5,349	24%	6%	53%	17
29	INDONESIA	92	9	75%	25%	0%	8
30	LUXEMBOURG	5,660	3,189	25%	13%	38%	8
31	PHILIPPINES	1,265	1,100	0%	25%	75%	8
32	POLAND	519	570	25%	50%	25%	8
33	SOUTH AFRICA	113	98	50%	50%	0%	8
34	BERMUDA	319	240	17%	83%	0%	6
35	ISRAEL	3,496	3,291	0%	50%	50%	6
36	RUSSIA	22	25	100%	0%	0%	4
37	PAKISTAN	73	102	33%	67%	0%	3
	Average	91,358	90,649	10%	18%	42%	

Among these six countries, the proportion of companies that could reduce emissions at $100 or less is the largest in China at a rate of 14%, followed by the United States at 10%, but this order changes at shadow prices of $1,000 and $10,000 or less. At $1,000 or less, Japan’s proportion becomes the second largest at a rate of 35%, slightly below China at 36%. At $10,000 or less, Japan’s proportion becomes the largest at a rate of 83%, followed by the United States and China, both at 74%. Although the number of observations in emerging countries is limited, the study found that a majority of companies in certain emerging countries can reduce emissions relatively inexpensively. For instance, 63% of the observed companies in Indonesia can reduce emissions at $20 or less, followed by 52% in Thailand and 35% in Malaysia. These price gaps among different countries indicate the economic rationale for an emissions trading scheme and the importance of the Clean Development Mechanism (CDM) to achieve emissions reductions on a global level.

In addition, a large disparity was found to exist between the average and median shadow prices, suggesting that the average price in each country is being supported by a set of companies and that many companies can reduce emissions relatively inexpensively.

The shadow price is the lowest for utilities companies at a median price at $46, followed by construction and material companies at $65 and basic resource companies at $184; by contrast, the price is the highest in the technology sector at $22,092 (Table 2). Shadow prices are relatively low in heavy industries such as construction and materials at $65 and basic resources at $184, and more than 50% of the observed companies in these industries can reduce emissions at a cost of $100 or less. Half of the observed companies in the oil and gas and chemicals industries can reduce emissions at a cost of $1,000 or less, whereas the shadow prices in the technology and media sectors are high–only a low percentage of the observed companies in these sectors can reduce emissions at a cost of $1,000 or less. If a cap on emissions were introduced at the same level in all industries, then those most readily affected would be industries with high shadow prices, such as technology and media, and those least affected would be industries with low shadow prices, such as utilities and basic resources.

**Table 2:** Shadow price by sector.

	Industry	Average Shadow Price (US$)	Median Shadow Price	Shadow Price (<100$)	Shadow Price (100$– 1,000$)	Shadow Price (1,001$–10,000$)	# obs.
1	Utilities	245	46	60%	32%	8%	308
2	Construction & Materials	2,315	65	52%	13%	31%	311
3	Basic Resources	456	184	37%	52%	10%	434
4	Oil & Gas	1,673	582	13%	53%	31%	375
5	Chemicals	2,020	766	10%	50%	37%	254
6	Food & Beverages	4,457	3,269	3%	13%	80%	328
7	Automobiles & Parts	6,969	3,491	3%	22%	60%	146
8	Travel & Leisure	4,060	4,200	5%	20%	72%	289
9	Industrial Goods & Services	11,478	4,811	2%	17%	46%	1,614
10	Retail, Financial & Real Estate	9,620	5,602	0%	6%	65%	547
11	Personal & Household Goods	16,819	8,718	0%	4%	49%	434
12	Telecommunications	14,235	11,044	0%	5%	38%	208
13	Healthcare	13,666	11,605	1%	3%	41%	387
14	Media	20,341	17,833	1%	0%	24%	285
15	Technology	29,399	22,092	0%	3%	29%	565
	Average	119,018	118,606	10%	18%	42%	

## Discussion

**Figure 1:**
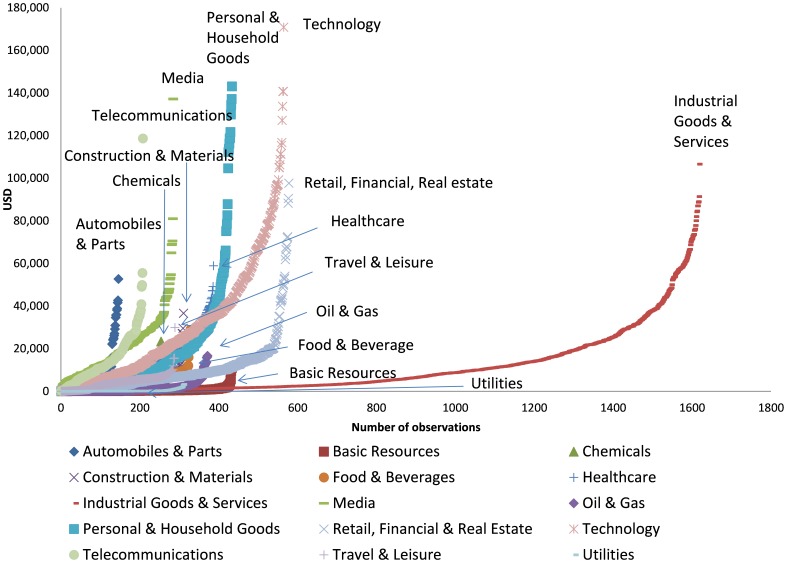
Shadow price by sector.

There are major disparities in the shadow price both among industries and within industries–some companies can reduce emissions at a relatively inexpensive price, whereas others cannot, even in the same industry (Figure 1). This disparity is particularly evident in industries such as technology, personal and household goods, and media. This finding supports the idea of a cap and trade emissions scheme because the variations in the shadow price for different companies suggests that it is economically efficient to trade carbon credits between companies with a low cost of emissions reductions and those with a high cost. The wide variation of shadow prices among companies within individual industries further indicates that an emissions trading scheme could be established not only among industries but also within industries; even if the system targets a single domestic sector, it is still effective. A shadow price that varies by company also assists in explaining which firms would become sellers and buyers of carbon credits and thus in determining the approximate quantities bought and sold in carbon markets. If the same level of carbon constraints (total quantity reduction) were to be imposed on all industries, then technology and other industries with high shadow prices would become buyers of carbon credits, and utilities and other industries with low shadow prices would become sellers. However, in reality, it is highly unlikely that the same level of emissions cap would be introduced for all industries, given their differing quantities of GHG emissions. As in the cap and trade system, it would be possible to estimate the tax burden on each business if an environmental tax were introduced.

Fig. 1 indicates that the shadow price of GHG emissions in the utility sector is much lower than in other sectors. The average shadow price of utility companies is $245 per ton of GHG, and the median is $46 per ton of GHG. The results reveal that 35% of the observed companies can reduce emissions at $20 per ton of GHG or less, 60% at $100 per ton of GHG or less, and 75% at $180 per ton of GHG or less. Furthermore, more than 40% of the observed companies in the United States can reduce emissions at $20 per ton of GHG or less. Moreover, a majority of companies in these five countries can reduce emissions at $100 per ton of GHG or less.

**Figure 2:**
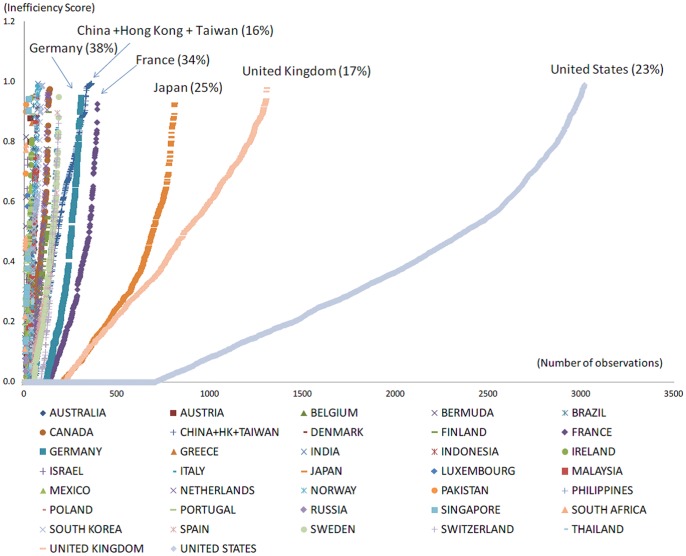
Inefficiency score by country.

Fig. 2 shows the operating efficiency (inefficiency score) by country in terms of the GHG emissions for 1,024 companies in 37 countries worldwide during the eight-year period from 2002 through 2009. The six countries shown in the figure are the United States, the United Kingdom, Japan, France, China (including Hong Kong and Taiwan), and Germany, all of which had large sample sizes; the number in parentheses is the frontier percentage (the percentage of results that were assessable as having an inefficiency score of zero and as conducting efficient operations) for each country.

Each country has companies with a high level of operating efficiency (companies with low inefficiency scores) and companies with low operating efficiency (companies with high inefficiency scores) (see Fig. 2). Because the data sample from the companies in the United States is the largest, the curve for the United States swings far to the right, but the frontier percentage clearly indicates that the United States does not simply have an especially high concentration of top-quality companies that would constitute the frontier curve. In fact, quality companies exist in every country in the world, and although quality companies may be concentrated in the center of the sample, the existence of frontier companies in China at a similar percentage relative to those in the United Kingdom indicates that any misconception of emerging market businesses being synonymous with inefficient businesses should be eliminated. Moreover, frontier companies and inefficient companies are not distributed in a manner that indicates that companies in advanced countries are synonymous with highly efficient companies or that emerging market companies are synonymous with inefficient companies. Rather, inefficient companies exist in every country, which suggests that a general improvement in such companies is required to reduce the world’s GHG emissions and to ensure continued economic growth (see Table S1 and Figure S3). To reduce GHG emissions in the world, policy measures are needed to advance operations, and such measures need to consider the GHG emissions of companies in specific countries or regions and the emissions in all countries.

### Policy Implications

Disparities in market competitiveness originate from the medium- to long-term management efforts of businesses, and eliminating these disparities is time consuming because it requires improvements in operating technology.

The figures and tables above indicate that there are major disparities in the shadow price, both among industries and within industries, similar to the disparities observed in market competitiveness. This finding appears to have extremely important implications concerning the administration of environmental issues, especially for the design and introduction of emissions trading schemes, as the research findings support the introduction of emissions trading schemes from all perspectives. Emissions trading schemes allow for the flexible fulfillment of GHG reduction obligations by establishing limits (gaps) for GHG emissions and allowing trading within those emissions limits. By allowing companies and groups with varying GHG reduction costs to trade emission rights according to their respective needs, inexpensive initiatives to reduce emissions will be selected efficiently, thus promoting the efficient reduction of emissions for society as a whole.

In this study, the shadow prices were found to vary greatly, both within and outside of industries. The disparities in shadow prices that were observed in this study clearly indicate that the introduction of emissions trading schemes would make it economically rational for companies with high shadow prices to trade emissions rights with companies with low shadow prices to promote the overall reduction of GHG emissions in an efficient manner in Japan and in other countries. Furthermore, the finding that shadow prices are widely divergent among individual companies, both across industries and within industries, indicates that emissions trading schemes could be established not only among industries but also within industries. In other words, emissions trading schemes could function meaningfully even if they target a single domestic industry.

## Supporting Information

Figure S1 **Shadow price of GHG emissions by country.**
(TIF)

Figure S2 **Mean and median value of the shadow price by region and by country.**
(TIF)

Figure S3 **Inefficiency score by region.**
(TIF)

Table S1 **Summary of inefficiency scores and the number of companies by country.**
(TIF)

Text S1 **Supporting Information.**
(DOC)
