# Supplementary figures and images for: The True Cost of Greenhouse Gas Emissions: Analysis of 1,000 Global Companies
Source: PLoS One. 2013 Nov 12;8(11):e78703. doi: 10.1371/journal.pone.0078703 (PMC3827104; doi:10.1371/journal.pone.0078703)

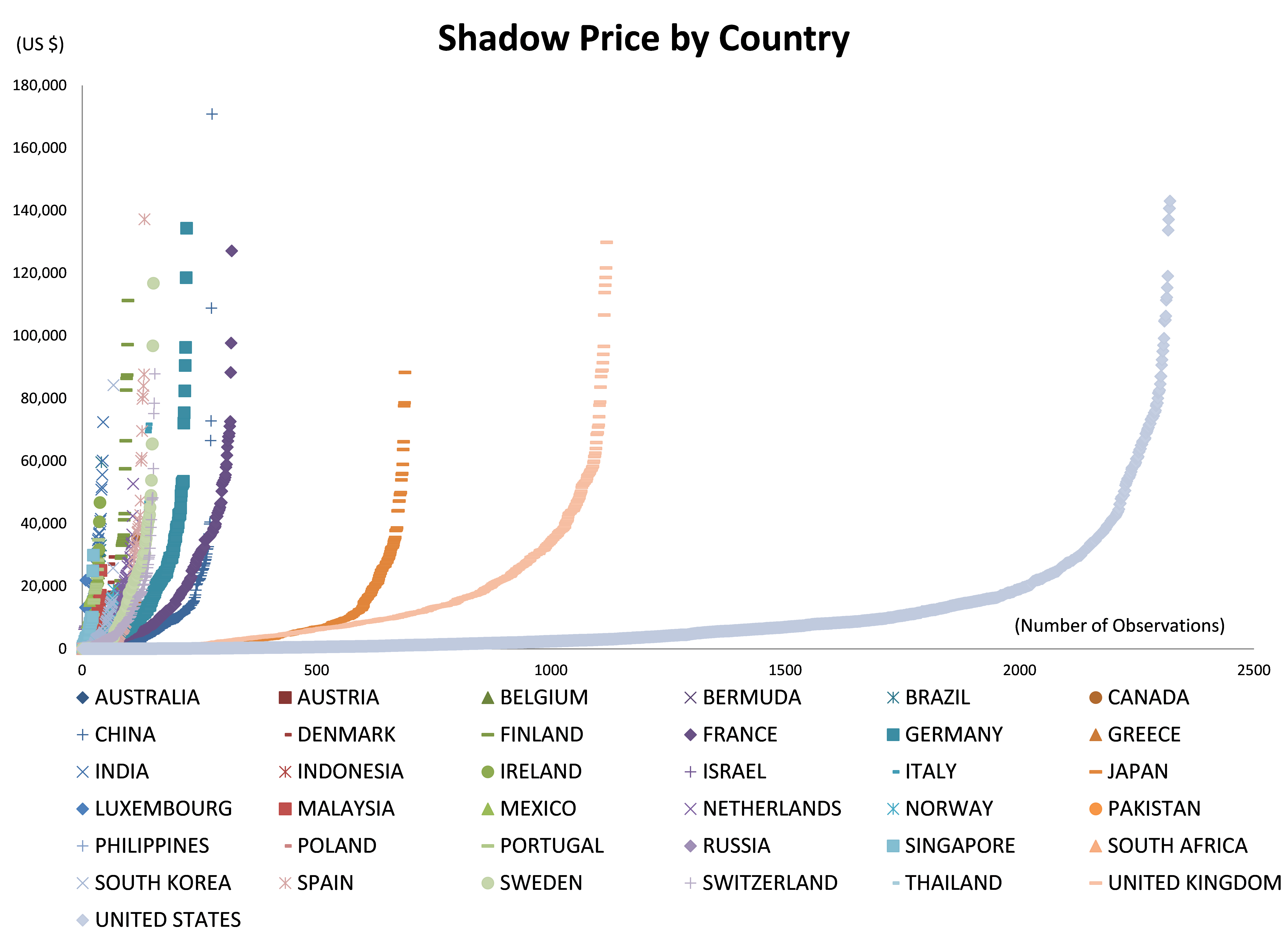

Supplement: Figure S1 — Shadow price of GHG emissions by country. (TIF) [file pone.0078703.s001.tif]

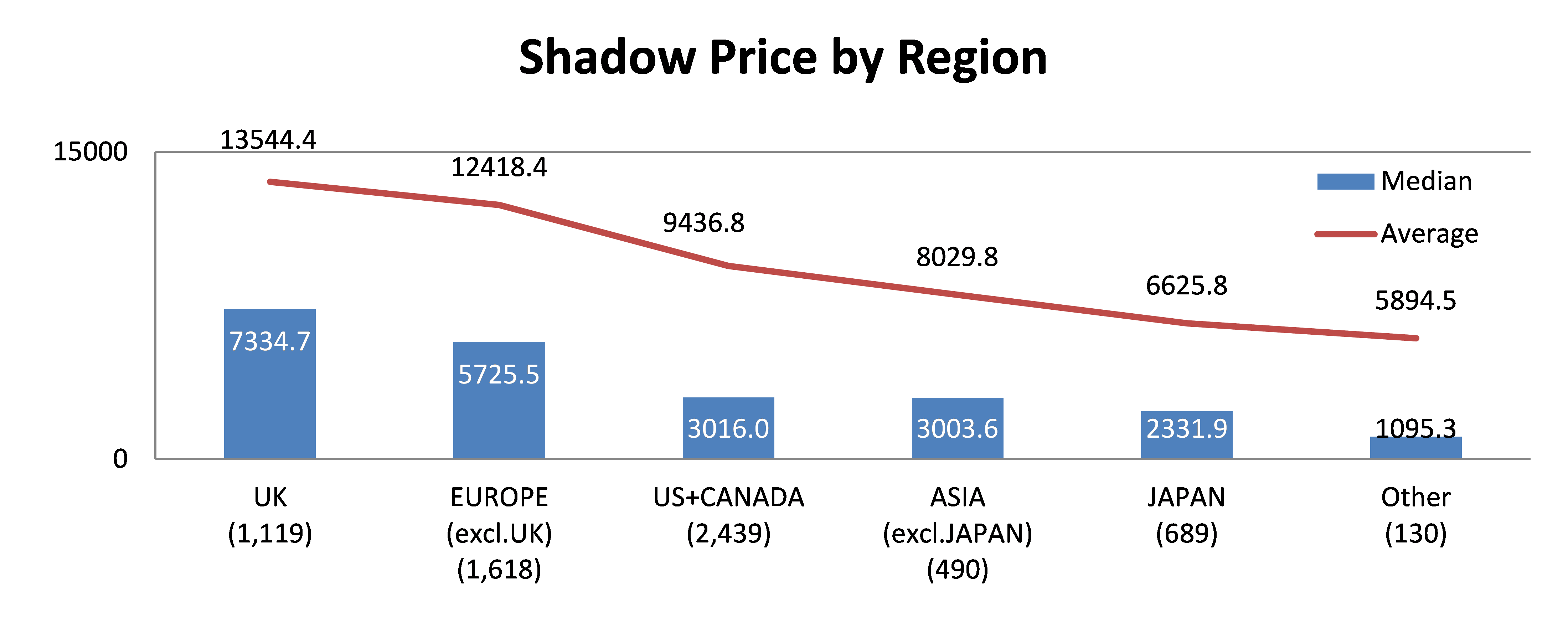

Supplement: Figure S2 — Mean and median value of the shadow price by region and by country. (TIF) [file pone.0078703.s002.tif]

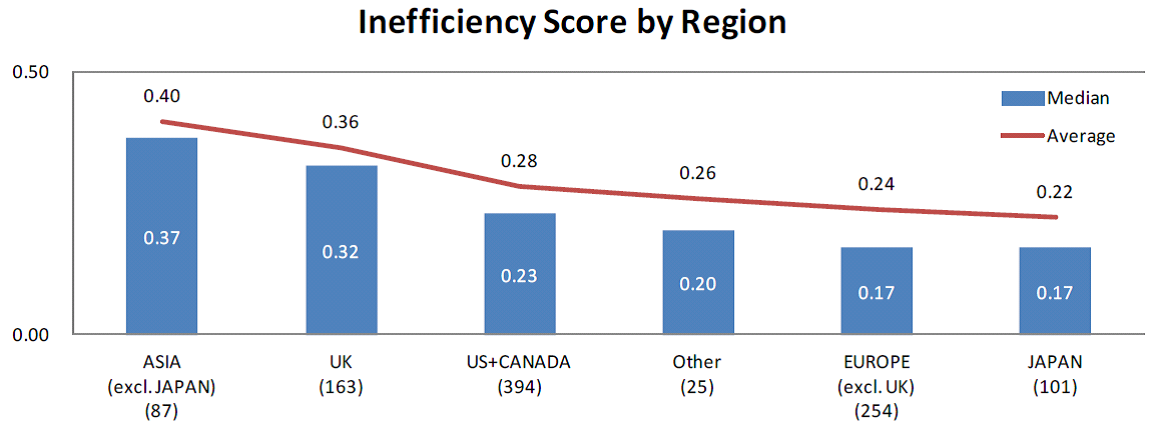

Supplement: Figure S3 — Inefficiency score by region. (TIF) [file pone.0078703.s003.tif]

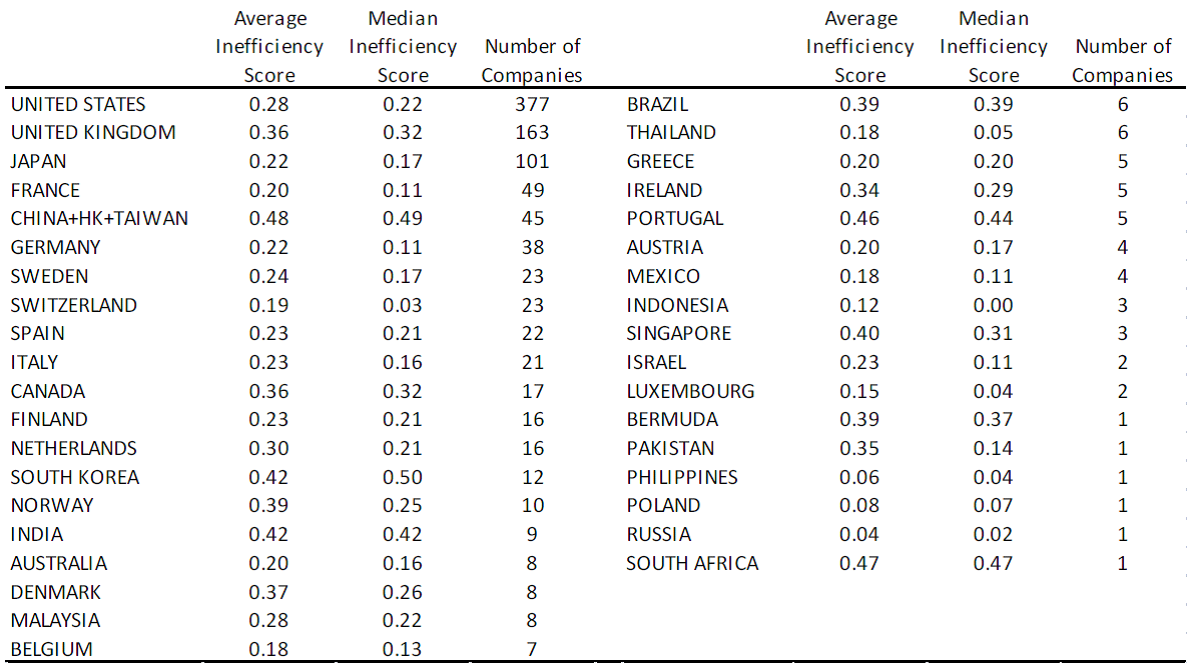

Supplement: Table S1 — Summary of inefficiency scores and the number of companies by country. (TIF) [file pone.0078703.s004.tif]
