# Supplementary material for: The True Cost of Greenhouse Gas Emissions: Analysis of 1,000 Global Companies
Source: PLoS One. 2013 Nov 12;8(11):e78703. doi: 10.1371/journal.pone.0078703 (PMC3827104; doi:10.1371/journal.pone.0078703)
Supplement: Text S1 — Supporting Information. (DOC) [file pone.0078703.s005.doc]

### Supplementary Information

Figure S1 shows the results of Table S1. The average value is summarized in Figure S2 by region and country. The numbers in parentheses in Figure S2 are the number of companies covered. Table S1 shows the average and median inefficiency scores and the number of companies by country for the above-mentioned eight-year period.

Table S1 indicates that the inefficiency scores for France, Germany, Switzerland, and other European countries, as well as Japan, are low, whereas the scores for the United Kingdom and China are high. In addition, the average inefficiency score is higher than the median in nearly all countries (33 out of 37 countries), which indicates that the average is being raised by a certain portion of the companies and that the companies conducting inefficient operations in each country represent a small percentage of the total.

Because the number of companies is limited in some countries, the above results are summarized by region in Figure S3. The numbers in parentheses in Figure S3 are the number of companies covered. In this figure, Asia (excluding Japan) consists of eight countries: China/Hong Kong/Taiwan, South Korea, India, Singapore, Thailand, Malaysia, Indonesia, and the Philippines. Europe (excluding the United Kingdom) in this figure includes 16 countries: Germany, France, Switzerland, Sweden, Spain, Italy, Finland, Norway, Denmark, Belgium, the Netherlands, Greece, Portugal, Ireland, Austria, and Luxembourg. Nine countries are considered: Australia, Brazil, Mexico, Israel, Russia, Poland, Pakistan, Bermuda, and South Africa.

These regional results indicate that the inefficiency scores by region decrease in the following order: Asia (excluding Japan), the United Kingdom, the United States/Canada, the other countries, Europe (excluding the United Kingdom), and Japan. A low inefficiency score indicates that high operating efficiency has been achieved from all perspectives, including the perspectives of capital, labor, materials, and GHG management. Japanese companies have thus far been globally recognized for their high level of environmental technologies and thoroughness in pursuing energy savings in their production lines, and this study has provided evidence that Japanese companies in all industrial categories report high levels of operating efficiency in terms of GHG emissions compared with companies in other countries. This efficiency illustrates the strength of Japan’s relative international competitiveness and suggests that if carbon constraints were imposed, Japan would experience a burden that is among the lightest of any country in the world.
